# Standardized Screening for Mental Health Needs of Detained Youths from Various Ethnic Origins: The Dutch Massachusetts Youth Screening Instrument-Second Version (MAYSI-2)

**DOI:** 10.1007/s10862-014-9476-4

**Published:** 2014-12-28

**Authors:** Olivier F. Colins, Thomas Grisso, Pauline Vahl, Laura Guy, Eva Mulder, Natasja Hornby, Christine Pronk, Monica Markus, Theo Doreleijers, Robert Vermeiren

**Affiliations:** 1 Department of Child and Adolescent Psychiatry, Leiden University Medical Center, Curium-LUMC, Endegeesterstraatweg 27, AK 2342 Leiden, The Netherlands; 2 Academic Workplace Forensic Care for Youth (Academische Werkplaats Forensische Zorg voor Jeugd), Postbus 94, 7200 AB Zutphen, Netherlands; 3 Department of Psychiatry, University of Massachusetts Medical School, l 55 Lake Avenue, North Worcester, MA 01655 USA; 4 Child And Adolescent Psychiatry, VU Medical Center Amsterdam, Postbus 303, 115ZG Duivendrecht, The Netherlands

**Keywords:** Self-report, Juvenile justice, Antisocial, Ethnicity, MAYSI-2, Mental health, Screening

## Abstract

In the U.S., the Massachusetts Youth Screening Instrument-Second Version (MAYSI-2) has been shown to be a reliable and valid tool to identify youth with mental health needs upon entry in detention facilities. The present study examined the factor structure, internal consistency, and convergent validity of the Dutch MAYSI-2 administered as part of routine clinical assessments in up to 955 detained male adolescents. Standardized mental health screening questionnaires (Youth Self-Report and Strengths and Difficulties Questionnaire) were used to test the convergent validity of the Dutch MAYSI-2. Confirmatory factor analyses showed that the factor structure of the original MAYSI-2 could be replicated with the Dutch MAYSI-2. Internal consistency indices showed that the Dutch MAYSI-2 provides a reliable screening of mental health needs. In addition, the Dutch MAYSI-2 scales were related with conceptually parallel measures of the same targeted mental health needs in the total group. With a few exceptions, the internal consistency and convergent validity was supported across ethnic groups as well. Overall, these results suggest the psychometric properties of the Dutch MAYSI-2 to be promising. Implications and limitations of the current study’s findings and directions for future research are discussed.

A substantial number of detained juveniles have mental health problems, including psychiatric disorders such as depression and trauma-related anxiety disorders (Colins et al. 2010; Vermeiren et al. 2006). In many countries, policy makers, researchers and clinicians (e.g., Wasserman et al. 2003) now recommend mental health screening for every youth being detained to determine the need for emergency mental health services to avert crises (e.g., suicide risk) and comprehensive assessment (e.g., to examine if symptoms are indicative of a psychiatric disorder). There are many methods for assessing mental health problems in juvenile justice settings (Grisso et al. 2005). However, most methods require more time and staff expertise than most youth detention centers can afford. Thus there has been a worldwide lack of reliable and efficient ways to identify youth with mental health needs upon entry to these facilities (e.g., Grisso et al. 2005; Harrington et al. 2005). The Massachusetts Youth Screening Instrument-Second Version (Grisso and Barnum 2006) was developed specifically to fill this void.

The MAYSI-2 was designed to identify youths who report symptoms of distress (e.g., depressed mood) or manifest feelings or behaviors (e.g., reported thoughts of suicide) that might require immediate intervention (such as suicide precautions) or might be in need of further assessment to determine whether they have a psychiatric disorder (Grisso 2007; Grisso et al. 2005). The MAYSI-2 requires only 15 min to administer and therefore can be easily used in detention facilities. MAYSI-2 subscale cut-off scores are used to identify youth in immediate crisis or at elevated risk for suicide. Research in the U.S. has demonstrated the MAYSI-2’s reliability and validity (Grisso et al. 2012), and its clinical usefulness for American juvenile detention facilities is supported by a growing number of studies showing, for example, that use of the MAYSI-2 increases staff referrals of detained youth to mental health counselors (Lopez-Williams et al. 2006), leads to more efficient identification of detained youth at risk for suicide (Williams et al. 2008), and helps to identify youths at risk for institutional misbehavior (Butler et al. 2007).

Given the lack of appropriate mental health screening in youth detention centers in the Netherlands (Youth Care Inspection 2007), the Dutch Ministry of Safety and Justice recently implemented the Dutch MAYSI-2 (Markus et al. 2009) as part of a standardized mental health screening process in all youth detention centers throughout the country. For several reasons, it is critical to examine whether the robust psychometric properties of the test generalize from American to Dutch samples. First, detention centers in the Netherlands are dealing with youths from diverse countries and cultures that differ from those typically seen in the U.S. (e.g., Moroccan versus African-American youths). Although, for example, the internal consistency of the MAYSI-2 scales and percentages of detained youths in the U.S. at or above MAYSI-2 caution cut-offs are very similar across ethnic groups (Vincent et al. 2008; Grisso et al. 2001), this may not be the case outside the U.S (e.g., Colins et al. 2013; Veen et al. 2010). A second reason to investigate the psychometrics of the Dutch MAYSI-2 relates to potential problems arising from its translation from English. Whenever a psychological test is translated, some words may not retain their intended meaning. Another language-related problem stems from the latent meaning of words. Even when accurately translated, some words (e.g., anxious) may produce differences in their associative meaning (Grisso 2012; Cauffman and MacIntosh 2006; McCoy 2010). A third justification for investigating the tool’s performance in a Dutch sample is related to potential differences between nations in judicial proceedings. Whereas mental health services in the Netherlands are widely available outside of detention settings (Grisso 2007; Vermeiren et al. 2006), adolescents in some U.S. communities are temporarily detained when more appropriate mental health services are not available (Grisso 2004). Accordingly, because youths in the U.S. may receive mental health care for the first time while in detention, a relatively larger proportion of detained youth in the U.S. potentially may be at or above MAYSI-2 cut-off scores compared with youth in the Netherlands. In light of different base rates of mental health problems, policies outlining how to use the MAYSI-2 as a screening tool may vary from country to country (Lennox et al. 2014).

The present study was designed to test the psychometric properties of the official Dutch version of the MAYSI-2. First, confirmatory factor analyses were performed to test if the factor structure of the MAYSI-2 could be replicated with the Dutch MAYSI-2. We are not aware of any previous studies in which the factor structure of the MAYSI-2 was investigated using a confirmatory factor analytical framework. Second, the internal consistency of the MAYSI-2 scales was examined. Based on research in the U.S. (e.g., Ford et al. 2008; Grisso et al. 2001; Grisso et al. 2012), it was expected that the internal consistency (at least as indexed by Cronbach’s alpha) would be lowest for the Depressed-Anxious, Thought Disturbance and Traumatic Experiences scales (i.e., .70 or lower). Third, the convergent validity of the Dutch MAYSI-2 was examined by testing whether MAYSI-2 scales were related to conceptually parallel scales from other screening tools commonly used in the Netherlands. It was expected that there would be significant positive associations between the MAYSI-2 subscales and conceptually similar scales on the Youth Self-Report (Achenbach 1991) and the self-report version of the Strengths and Difficulties Questionnaire (Goodman 1997).

## Method

### Participants

The sample comprises male adolescents who entered one of two large youth detention centers (YDCs) in the Netherlands between May 2008 and March 2012. Data for 1250 detained male adolescents who took the MAYSI-2 for the first time were made available to the authors. For several reasons, 187 youths were excluded from the current study. First, as of January 2010, civil-law involved juveniles (i.e., Youth Care Plus youths) can no longer be confined in a youth detention center in the Netherlands. We therefore excluded the 109 civil-law youths who were screened and assessed between May 2008 and January 2010. Second, we did not use data from one male adolescent administered the MAYSI-2 between May 2008 and January 2010 because it was unclear whether he was referred in the context of penal or civil law. Third, 64 boys were excluded because data about their ethnic origin was missing. Finally, 121 boys were excluded because they were older than 18 years, and thus, exceeded the age range for which the MAYSI-2 was developed.^1^ The current study, therefore, used mental health screening data from up to 955 detained male adolescents. Of the 955 youths who completed the MAYSI-2 and the Strengths and Difficulties Questionnaire (SDQ), 368 also were administered to the Youth Self-Report (YSR) and the substance use related questions within 2 weeks after being administered the MAYSI-2.

The mean age of our sample (*n* = 955) was 16.45 years (*SD* = 1.06; range 12.59 to 17.99) with 11.1 % of the boys aged 12 to 14 and 88.9 % aged 15 to 17. Approximately 90 % of the participants were detained while awaiting final trial (pretrial), whereas the remaining 10 % were detained by following conviction. With regard to ethnicity, 22.2 % of the sample was from Dutch origin, 27.0 % from Moroccan origin, 21.7 % from Antillean/Surinamese origin and 29.1 % from other origins (e.g., Turkish). In addition, 39.5 % of the boys had been detained in the past (mean number of past detentions = 0.75; *SD* = 1.31; range = 0 to 8). The detention subsample for whom YSR data were available (*n* = 368) was not significantly different from the full sample (*n* = 955) with regard to age, ethnicity, number of times being detained in the past, MAYSI-2 scale scores and SDQ-scales scores (details are available upon request from the first author).

### Measures

#### Massachusetts Youth Screening Instrument-Second Version (MAYSI-2)

The MAYSI-2 (Grisso and Barnum 2006) is a 52 yes/no item screening tool on which youths report the presence or absence of symptoms or behaviors related to several areas of emotional, behavioral, and psychological disturbances experienced “within the past few months.” The MAYSI-2 was developed and normed for administration by non-clinicians to youth aged 12–17 years when entering a juvenile justice setting. The MAYSI-2 can be administered in about 15 min by computer or paper and pencil, with little difference between these two forms in the scores obtained (Hayes et al. 2005). Factor analyses indicated that the items produce scores on six clinical scales: Alcohol-Drug Use, Angry-Irritable, Depressed-Anxious, Somatic Complaints, Suicide Ideation, and Thought Disturbance (for boys only); and one non-clinical scale (Traumatic Experiences) that screens for reported exposure to potentially traumatic events (Grisso and Barnum 2006). A few items on the MAYSI-2 questionnaire do not contribute to any of the scales but were retained for research and/or clinical purposes. There is no MAYSI-2 total score as the test was not intended to measure a broader construct such as mental distress or emotional disturbance (Grisso and Barnum 2006). Each of the six clinical MAYSI-2 scales has a “caution” cutoff developed by comparing the particular scale to conceptually comparable scales on the Millon Adolescent Clinical Inventory (MACI; Millon and Davis 1993) and the Youth Self-Report (YSR; Achenbach 1991). Specifically, for each MAYSI-2 scale, the developers identified the score that most closely overlapped with the “clinical significance” cutoff scores of parallel scales on the MACI and YSR. Youths scoring above a MAYSI-2 caution cutoff would most likely score high on similar tests of adolescent disturbances, and therefore might be in need of clinical attention. Each clinical scale also has a “warning” cutoff identifying scores obtained by the top 10 % of youths in the original Massachusetts normative sample, flagging youths who are even more in need of clinical attention. The MAYSI-2 manual also encourages clinicians to do a ‘second screening’ to obtain information to assess whether the young person obtained the high score for the reasons that the scale intends to measure. For example, a young person might have scored high on Suicide Ideation, yet the second screening questions reveal that the young person was referring to a period of time 2 months ago, and that he has never thought about it since then. The official Dutch version of the MAYSI-2 was developed in 2008 using translation back-translation procedures (Markus et al. 2009).

#### Youth Self-Report (YSR)

The Dutch YSR (Verhulst et al. 1997 a) consists of eight “competence” items (not administered in this study) and 118 “problem” items that youth answer on a three-level *Likert*-type scale as being not true, sometimes true, or very true for themselves. The responses to the problem items contribute to eight narrow-band scales that identify problem areas (Withdrawn/Depressed, Somatic Complaints, Anxious-Depressed, Social Problems, Thought Problems, Attention Problems, Delinquent Behavior and Aggressive Behavior), and two broadband scales (Internalizing and Externalizing). For males only, several items also contribute to a Self-Destruction scale. Similar to Grisso (Grisso et al. 2001), we considered five of the Dutch MAYSI-2 scales to be conceptually parallel scales in the YSR, and we therefore used them to examine the convergent validity of the Dutch MAYSI-2. These scales were (MAYSI-2 and YSR, respectively): Angry-Irritable and Aggressive Behavior; Depressed-Anxious and Anxious-Depressed; Somatic Complaints and Somatic Complaints; Suicide Ideation and Self-Destruction; and Thought Disturbance and Thought Problems. Therefore the current study will only use these five YSR subscales.).

The *Strength and Difficulties Questionnaire* (SDQ). The SDQ (Goodman 1997) is a screening instrument for psychosocial functioning of children and adolescents. The SDQ has four difficulty subscales (Hyperactivity, Conduct Problems, Peer Problems, Emotional Symptoms) and one strength subscale (Prosocial Behavior). Each subscale consists of five items with three response categories (not true = 0, somewhat true = 1, certainly true = 2). For testing the Dutch MAYSI-2’s convergent validity, we used two scales of the Dutch SDQ Self-Report version (van Widenfelt et al. 2003) that conceptually parallel two MAYSI-2 scales: MAYSI-2 Angry-Irritable – SDQ Conduct Problems; and MAYSI-2 Depressed-Anxious- SDQ Emotional Symptoms.

#### Diagnostic-Interview Schedule for Children-Fourth Version (DISC-IV)

Neither the YSR nor the SDQ includes a subscale assessing alcohol and substance use. To study the convergent validity of the MAYSI-2 Alcohol/Drug Use subscale, we used items from the Substance Use Disorder (SUD) Module of the DISC-IV (Shaffer et al. 2000). Because the MAYSI-2 Alcohol/Drug Use subscale is intended to identify youths at risk to develop or to have one or more substance use disorders, we created a DISC-IV variable that reflects intense use of alcohol and marijuana, the most commonly used drug in detained male adolescents (Colins et al. 2009; Colins et al. 2010). Based on previous DISC-IV research in detained adolescents (Colins et al. 2009), we first created two dichotomous variables: past year intense alcohol use and past year intense marijuana use. Past year intense alcohol use refers to having drunk alcohol every week in the past year and reflects the highest score the youths can have on this particular DISC-IV question. Past year intense marijuana use refers to a period in the past year when participants used marijuana on a weekly basis (i.e., between one to 2 days a week and almost every day). Second, and because the MAYSI-2 Alcohol-Drug Use subscale does not differentiate between alcohol and substance use, we created the dichotomous variable ‘Intense Alcohol-Marijuana Use’. Youths who reported past year intense use of alcohol and/or marijuana were identified as intense alcohol-marijuana users.

#### Ethnic Background

Based on the Dutch standard classification of ethnic groups (Central Bureau Of Statistics 2012), a participant was categorized as “Moroccan” or “Antillean or Surinamese” when the adolescent himself and/or at least one parent had been born in Morocco or Dutch Antilles or Surinam, respectively. When both parents were of different non-Dutch origin, we used the mother’s country of birth to determine the child’s ethnicity. Participants were classified as Dutch when both parents and the child were born in the Netherlands. All other participants were assigned to the “Mixed” origin group (subsequently referred to as “Mixed boys” group).

### Procedure

Youths were administered the Dutch MAYSI-2, SDQ, YSR and DISC-IV as part of routine mental health screening in two youth detention centers in the Netherlands. Between May 2008 and July 2010, only the Dutch MAYSI-2 and the SDQ were administered on a stand-alone computer in the presence of non-clinical youth detention center personnel to all youth within a few days after detention entry. From July 2010 to March 2012 additional mental health screening and assessment measures (e.g., the YSR, the DISC-IV) were introduced and administered to all youth within the first couple of weeks after being administered the Dutch MAYSI-2 and SDQ. Trained masters level students and test assistants with a master’s degree administered these additional instruments to each youth. The MAYSI-2 and SDQ were administered on average 3.54 days after detention intake (*SD* = 4.01; range 0 to 67 days; median = 3 days), with 76.7 % of participants having been administered these mental health screening instruments within 4 days and 92.1 % within 7 days after detention intake. The Dutch YSR (*n* = 396) was administered on average 2.37 days after the Dutch MAYSI-2 (*SD* = 2.37; range, 0 to 13; median = 2.00 days). Youths were aware that mental health screening was part of the YDCs’ routine and that mental health screening outcomes were available to youth detention center personnel. Through standardized information provided by the youth detention personnel upon the start of detention, youths and their parents/caretakers were informed that mental health screening outcomes would be used -unless they refused- for scientific research. The Medical Ethical Review Board of the Leiden University Medical Center certified that our study meet the Dutch law of behavioral research because all data were derived as part of the clinical assessment.

### Overview of Statistical Analyses

First, confirmatory factor analyses were performed to test the factor structure of the Dutch MAYSI-2 (estimator: Robust Weighted Least Squares; software program: Mplus 6). Model fit was assessed using *χ* ^2^, Root Mean Square Error of Approximation (RMSEA), and Comparative Fit Index (CFI). With regard to *χ* ^2^, a good fit is indicated when *χ* ^2^/df ≤ 2, whereas *χ* ^2^/df ≤ 3 is indicative of an acceptable fit (Schermelleh-Engel et al. 2003). RMSEA scores below .05 indicate good fit, whereas scores between .05 and .08 indicate acceptable fit. A CFI score of .95 or above indicates excellent fit, and a CFI score of .90 to .94 indicates good fit (Hu and Bentler 1999). Second, the reliability indices Cronbach’s alpha (α) and the mean corrected item-to-total correlation (MCITC) were computed. We considered α coefficients < .60 as insufficient, from .60 to .69 as marginal, from.70 to .79 as acceptable, from .80 to .89 as good, and above .90 as excellent (Barker et al. 1994). We used the Feldt test to compare the magnitude of the *α*s in the U.S. sample with the *α*s in the total Netherlands sample. To be acceptable, the MCITC should be above the recommended value of .30 (Nunnally and Bernstein 1994). Third, Dutch MAYSI-2 scale score distributions were examined by investigating means (SD) and the number of youths who scored at or above the U.S. MAYSI-2 caution and warning cut-offs. Chi-square statistics were used to test whether the proportions of Dutch youth in the total sample were significantly different (at *p* < 0.01) from the proportions of youths in the U.S. sample at or above these cut-offs (Grisso and Barnum 2006). Fourth, Pearson correlation coefficients (*r*) were used to assess the strength of the relation between the Dutch MAYSI-2 subscales and conceptually parallel SDQ and YSR subscales. We present point-biserial correlation coefficients (*r*_pb_) to examine the strength of the unique relation between the (continuous) Dutch MAYSI-2 Alcohol-Drug Use scale and (dichotomous) DISC-IV based Intense Alcohol-Substance Use. Finally, to better understand the relation between the Dutch MAYSI-2 scales and their conceptually parallel YSR scales, we performed a series of five multivariate regression analyses with all five YSR scales entered simultaneously as the independent variable and one Dutch MAYSI-2 scale as dependent variable. Partial correlation coefficients were computed to examine the unique relation between each YSR scale and a particular Dutch MAYSI-2 scale.

## Results

### Confirmatory Factor Analyses

The model specified that the 44 items load on the seven latent constructs (seven Dutch MAYSI-2 scales) as described in the MAYSI-2 manual (Grisso and Barnum 2006) and that these seven constructs were allowed to be correlated. The model indicated good fit according to one index (RMSEA = 0.046) and was just above or below the recommended cut-offs for acceptable fit according to the other two indices (*χ* ^2^ ^(2621.59)^/df ^(878)^ = 3.01; CFI = 0.86). Modification indices showed that allowing some items to correlate with each other improved the model fit (RMSEA =0.039; *χ* ^2^ ^(2118.07)^/df ^(862)^ = 2.46; CFI = 0.895).^2^ The factor loadings of this modified model are presented in the Appendix. Because of the small sample sizes in the ethnic subgroups, we did not test measurement invariance of the Dutch MAYSI-2 across these groups.

### Descriptive Information

Dutch MAYSI-2 scale mean scores and percentages of boys at or above the U.S. caution and warning cut-off are presented in Tables 1 and 2. *Z*-tests for proportions showed that the percentages of our total sample at or above the U.S. caution and warning cut-off were significantly lower than in the U.S. total sample (Table 2). Because between-group comparison in the absence of evidence of measurement invariance may not be meaningful and should be interpreted with caution, we did not test whether there were significant differences between the four ethnic groups, and between each of these four ethnic groups and the U.S. sample.

**Table 1:** Means (Standard Deviations) for MAYSI-2 scales in the total sample and by ethnic group

	Alcohol/Drug Use		Angry-Irritable		Depressed-Anxious		Somatic complaints		Suicide ideation		Thought disturbance		Traumatic experiences	
	M	(SD)	M	(SD)	M	(SD)	M	(SD)	M	(SD)	M	(SD)	M	(SD)
Total Sample (*n* = 955)	1.19	(1.96)	1.96	(2.15)	1.17	(1.50)	1.74	(1.47)	0.24	(0.80)	0.32	(0.69)	1.51	(1.39)
Dutch (*n* = 212)	2.10	(2.43)	2.64	(2.43)	1.31	(1.46)	1.98	(1.44)	0.38	(0.97)	0.39	(0.75)	1.77	(1.41)
Moroccan (*n* = 258)	0.47	(1.30)	1.31	(1.91)	0.84	(1.39)	1.47	(1.50)	0.13	(0.63)	0.22	(0.64)	1.15	(1.32)
Antil/Sur (*n* = 207)	1.28	(1.85)	2.18	(2.12)	1.28	(1.43)	1.74	(1.39)	0.26	(0.85)	0.40	(0.74)	1.60	(1.37)
Mixed (*n* = 278)	1.11	(1.85)	1.87	(1.98)	1.29	(1.63)	1.80	(1.49)	0.22	(0.74)	0.30	(0.65)	1.58	(1.39)

*Antil/Sur* = Antillean/Surinamese

**Table 2:** Percentages of youths At or above the U.S. MAYSI-2 caution and warning cut-off scores (total sample and by ethnic group)

	U.S. 2006 Sample		Total (*n* = 955)		Dutch (*n* = 212)		Moroccan (*n* = 258)		Antil/Sur (*n* =207)		Mixed (*n* = 278)	
Dutch MAYSI-2 Scales	Ca	Wa	Ca	Wa	Ca	Wa	Ca	Wa	Ca	Wa	Ca	Wa
Alcohol/Drug Use (4/6)	28	14	15.1	5.3	30.2	12.7	5.8	1.2	12.6	4.8	14.0	4.0
Angry-Irritable (5/8)	33	8	14.8	2.0	24.5	4.2	8.5	1.6	15.9	1.4	12.2	1.1
Depressed-Anxious (3/6)	30	7	16.6	1.9	21.2	0.5	10.5	1.2	14.0	1.9	20.9	3.6
Somatic complaints (3/6)	37	4	28.5	1.9	36.8	1.4	20.9	3.1	30.4	0.0	27.7	2.5
Suicide ideation (2/3)	15	10	5.4	3.4	9.0	5.7	2.7	1.6	6.3	3.9	4.7	2.9
Thought disturbance (1/2)	35	12.8	22.8	6.5	27.4	8.5	15.1	3.9	29.0	8.7	21.9	5.8
At or above at least 1 scale	68	31.6	51.0	14.9	64.6	24.1	32.6	7.8	57.5	15.0	52.9	14.4
At or above at least 2 scales	46	13.5	26.8	4.0	41.5	6.1	15.1	1.9	26.1	3.9	27.0	4.3

*Anti/Sur* = Antillean/Surinamese; *Ca* = Caution; *Wa* = Warning; Number between parentheses refers to the U.S. caution/warning cut-off; Percentages at or above caution also included the percentages at or above warning cut-off; * significantly different (*p* < 0.01) from percentages presented in the U.S. 2006 manual for males (*n* = 54,604)

### Internal Consistency

Table 3 shows that the *αs* for the Dutch MAYSI-2 scales in the total sample ranged from .61 to .85 with the exception of Thought Disturbance (α = .48) and Somatic Complaints (α = .59). The αs for various ethnic groups were substantially similar for all but two Dutch MAYSI-2 subscales (see Table 3). The Feldt test did not reveal significant differences between the αs from the U.S. sample and the total sample. MCITCs (Table 3) ranged from .33 to .60, and were, thus, all at or above the recommended value of .30, except for Thought Disturbance (.28). MCITCs were all above .30 for Dutch, Moroccan, and Mixed boys, except for Thought Disturbance in Dutch and Mixed boys (.25 and .22, respectively), and Depressed-Anxious in Dutch boys (.28). In Antillean/Surinamese boys, the MCITC values were below .30 for Depressed-Anxious and Thought Disturbance.

**Table 3:** Reliability Indices for MAYSI-2 scales for total sample and by ethnic group

MAYSI-2 scale (number of items in scale)	Total Sample (U.S.)^a^ (*n* = 955)		Dutch (*n* = 212)		Moroccan (*n* = 258)		Antil/Sur (*n* =207)		Mixed (*n* = 278)	
	*α*	*MC*	*α*	*MC*	*α*	*MC*	*α*	*MC*	*α*	*MC*
Alcohol/Drug Use (8)	.85 (.85)	.58 (.58)	.85	.59	.85	.58	.80	.51	.83	.56
Angry-Irritable (9)	.77 (.80)	.45 (.49)	.79	.48	.79	.48	.73	.41	.71	.40
Depressed-Anxious (9)	.64 (.72)	.33 (.40)	.59	.28	.70	.39	.56	.26	.68	.36
Somatic Complaints (6)	.59 (.75)	.34 (.49)	.54	.30	.67	.41	.54	.30	.59	.40
Suicide Ideation (5)	.80 (.80)	.60 (.60)	.80	.60	.84	.65	.83	.63	.77	.55
Thought Disturbance (5)	.48 (.61)	.28 (.37)	.46	.25	.61	.40	.41	.22	.45	.25
Traumatic experiences (5)	.61 (.63)	.37 (.39)	.59	.35	.64	.41	.56	.33	.59	.35

^a^Number in parentheses represents the value reported in the U.S. boys sample (Grisso, et al., 2002); Note: *α* = Alpha; *Antil/Sur* = Antillean or Surinamese; *MC* = mean corrected-item-to-total correlation; an inspection of the alpha-if-deleted output showed that deleting an item would not substantially be improved

Correlations among Dutch MAYSI-2 scales for the total sample ranged from .19 to .59 (mean *r* = .37) and closely approximated the mean intercorrelation of .39 among boys in the U.S. sample (Grisso and Barnum 2006). Correlations among Dutch MAYSI-2 scales for Dutch boys ranged from.13 to .63 (mean *r* = .35), for Moroccan boys from .15 to .65 (mean *r* = .47), for Antillean/Surinamese boys from .11 to .53 (mean *r* = .30), and for Mixed boys from .16 to .55 (mean *r* = .33).

### Construct Validity^3^

#### Youth Self-Report

Four of the five YSR scales were more highly correlated with their parallel MAYSI-2 scales than with any other Dutch MAYSI-2 scales (Table 4). The only exception was for the YSR Self-Destruction scale, which was more strongly related to the Dutch MAYSI-2 Angry-Irritable (*r* = .44) and Depressed-Anxious (*r* = .51) scales than its conceptually parallel Dutch MAYSI-2 Suicide Ideation scale (*r* = .36). Also, the correlation between YSR Thought Problems and Dutch MAYSI-2 Thought Disturbance (*r* = .49) was almost identical to the correlation between YSR Thought Problems and the Dutch MAYSI-2 Angry-Irritable (*r* = .43) and Depressed-Anxious (*r* = .48) scales. Results for the Dutch MAYSI-2 Angry-Irritable, Depressed-Anxious, Somatic Complaints and Thoughts Disturbance scales remained substantially similar when analyses were repeated for youths from ethnic subgroups (Table 4). However, in some ethnic groups the YSR Thought Problems scale was at least as strongly related to other MAYSI-2 scales as to its corresponding MAYSI-2 Thoughts Disturbance scale.

**Table 4:** Bivariate correlations of MAYSI-2 scales with conceptually parallel scales of youth self-report

	Total sample					Dutch					Moroccan					Antillean/Surinamese					Mixed				
MAYSI-2\ YSR	AB	AD	SC	SD	TP	AB	AD	SC	SD	TP	AB	AD	SC	SD	TP	AB	AD	SC	SD	TP	AB	AD	SC	SD	TP
Angry-Irritable	.66*	.34*	.30*	.44*	.43*	.69*	.41*	.17	.45*	.33*	.68*	.24	.28*	.37*	.37*	.60*	.25	.34*	.43*	.51*	.53*	.30*	.35*	.37*	.44*
Depressed-Anxious	.35*	.58*	.41*	.51*	.46*	.47*	.63*	.33*	.56*	.47*	.37*	.60*	.47*	.49*	.55	.28*	.53*	.35*	.49*	.25	.23	.54*	.41*	.50*	.48*
Somatic Complaints	.24*	.42*	.50*	.29*	.36*	.18	.31*	.48*	.08	.23*	.30*	.43*	.49*	.31*	.55*	.23	.47*	.54*	.44*	.19	.18	.41*	.45*	.34*	.36*
Suicide Ideation	.16*	.30*	.21*	.36*	.29*	.15	.25	.28*	.33*	.28*	.00	.53*	.44*	.36*	.49*	.13	.32*	.18	.36*	.27	.14	.33*	.08	.36*	.23
Thought Disturbance	.28*	.37*	.35*	.35*	.49*	.29*	.44*	.37*	.30*	.48*	.24	.53*	.48*	.36*	.51*	.44*	.43*	.35*	.47*	.32*	.26*	.30*	.23	.41*	.54*

Based on *n* = 368 (Dutch *n* = 89; Moroccan: *n* = 103; Antillean/Surinamese: *n* = 76; Mixed: *n* = 100); YSR = Youth Self-Report; AB = Aggressive Behavior; AD = Anxious/ Depressed; SC = Somatic Complaints =; SD = Self-destructive Behaviour; TP = Thought Problems; Bold figures indicate correlations between conceptually ‘parallel’ scales; * significant at *p ≤* 0.01 level (2-tailed)

Next, we performed a multivariate regression analysis with all five YSR scales entered simultaneously as independent variables and one Dutch MAYSI-2 scale as the dependent variable. In the total sample, only the conceptually parallel YSR scale was significantly correlated with the corresponding Dutch MAYSI-2 scale (Table 5). This series of multivariate regression analyses was repeated for each ethnic group. In Dutch boys the YSR Self-Destruction and Thought Problems scales were not significantly related with their parallel Dutch MAYSI-2 scales, although there was a tendency towards statistical significance that was set at *p* < .01 (Suicide Ideation: *p* = .10; Thoughts Disturbance; *p* = .02). In Moroccan boys, only the YSR scales Aggressive Behavior and Anxious-Depressed were related to their parallel Dutch MAYSI-2 scales. In Antillean/Surinamese boys, the YSR Anxious-Depressed and Thought Problems scales were not significantly related with their parallel Dutch MAYSI-2 scale. In boys from Mixed origins, the YSR Anxious-Depressed and Self-Destruction scales were not significantly related to their parallel Dutch MAYSI-2 scales.

**Table 5:** Partial correlations with five Youth Self-Report (YSR) scales as independent variables and one MAYSI-2 scales as dependent variable

	Total Sample					Dutch					Moroccan					Antillean/Surinamese					Mixed				
MAYSI-2\YSR	AB	AD	SC	SD	TP	AB	AD	SC	SD	TP	AB	AD	SC	SD	TP	AB	AD	SC	SD	TP	AB	AD	SC	SD	TP
Angry-Irritable	.54*	−.05	.08	.11	.07	.59*	.13	−.06	.13	−.13	.62*	−.07	.13	.05	.07	.44*	−.17	.13	.27	.22	.39*	−.08	.16	.10	.12
Depressed-Anxious	.08	.28*	.08	.09	.09	.21*	.35*	−.04	.11	.03	.23	.31*	.11	.03	.13	.06	.25	.04	.13	−.06	−.07	.19	.08	.16	.20
Somatic complaints	.08	.18*	.33*	−.11	.04	.10	.23	.43*	−.31*	−.01	.17	.10	.19	−.13	.27*	.02	.13	.37*	.10	−.10	−.01	.15	.26*	−.01	.06
Suicide ideation	−.05	.04	.001	.19*	.09	−.04	−.05	.13	.18	.17	−.22	.25	.10	.04	.24	−.08	−.11	−.04	.33*	.05	−.03	.13	−.16	.19	.07
Thought disturbance	.04	.05	.09	.01	.29*	.07	.18	.11	−.14	.26	.11	.26*	.17	−.07	.14	.14	−.10	.24	.02	.25	−.05	−.14	−.11	.25	.44*

*Note*: Based on n = 368 (Dutch *n* = 89; Moroccan: n = 103; Antillean/Surinamese: *n* = 76; Mixed: *n* = 100); AB = Aggressive Behavior; AD = Anxious/ Depressed; SC = Somatic Complaints; SD = Self-destructive behaviour; TP _=_ Thought Problems; Bold figures indicate correlations between conceptually ‘parallel’ scales; * significant at *p ≤* .01 level (2-tailed)

#### Strength and Difficulties Questionnaire

Both the SDQ Conduct Problems and Emotional Symptoms scales were more strongly correlated with their parallel Dutch MAYSI-2 scales than with any of the other Dutch MAYSI-2 scales (Conduct Problems to Angry-Irritable/Depressed-Anxious: *r* = .64/.36; Emotional Symptoms and Angry-Irritable/Depressed-Anxious: *r* = .36/.60). Multivariate analyses again showed that both the SDQ Conduct Problems and Emotional Symptoms scales were more strongly correlated with their parallel Dutch MAYSI-2 scales than with any of the other Dutch MAYSI-2 scales (Conduct Problems and Angry-Irritable/Depressed-Anxious: *r* = .60/.23; Emotional Symptoms and Angry-Irritable/Depressed-Anxious: *r* = .24/.56). These findings were substantially similar in each ethnic group (details available from the first author).

#### DISC-IV Alcohol and Marijuana Use

For the total sample, the Dutch MAYSI Alcohol/Drug Use scale was more strongly (*r*_pb_ = .54; *p* < .001) related to intense alcohol and marijuana use than to any other Dutch MAYSI-2 scale (*r*_pb’s_ below .17, except Suicide Ideation = .24). In addition, of the Dutch MAYSI-2 scales, the Alcohol/Drug Use scale had the strongest association with intense alcohol and marijuana use in Dutch (*r*_pb_ = .47; *p* < .001), Moroccan (*r*_pb_ = .29; *p* < .001), Antillean/Surinamese (*r*_pb_ = .59; *p* < .001), and Mixed boys (*r*_pb_ = .53; *p* < .001).

## Discussion

This is the first study to report on the psychometric properties of the Dutch MAYSI-2. Confirmatory factor analyses showed that the factor structure of the original MAYSI-2 could be replicated in detained male adolescents in the Netherlands. Relevant indices suggested that the Dutch MAYSI-2 provides an internally consistent method of screening mental health needs among detained boys. Although the commonly used αs were sometimes below the recommend cut-off, the alternative fit index that is less sensitive to the number of items in a given Dutch MAYSI-2 scale was most often indicative of at least acceptable internal consistency. Bivariate and partial correlation coefficients showed that the Dutch MAYSI-2 scales were strongly and only related with conceptually parallel scales of other tools, providing support for the Dutch MAYSI-2’s convergent validity. Yet, it must be acknowledged that the reliability and the convergent validity of some Dutch MAYSI-2 scales were not always equally well supported in all ethnic groups.

To test if the factor structure of the MAYSI-2 could be replicated when being administered in a country other than the U.S and in a different language, a confirmatory factor analysis was performed. Overall, the model fit indices showed that the factor structure received acceptable to good fit. Although the CFI was slightly below the recommend cut-off for acceptable fit, it has been argued that the adequacy of the CFI for evaluating model fit with large numbers of categorical items (e.g., yes versus no responses) has not been fully determined, suggesting CFI can be considered an ancillary measure of fit (Dedrick et al. 2008). This may explain why in previous studies that reported good or excellent model fit (according to the RMSEA) for the factor structure of mental health screening tools, CFI was not reported (e.g., Dumenci et al. 2004). Notwithstanding that the model fit is not excellent according to the commonly used recommended cut-offs, we argue that the model fit is sufficient enough, and are in line with fit indices that are reported for alternative screening tools (e.g., Dedrick et al. 2008). We do acknowledge that modification indices may be sample specific, and that it remains to be seen whether the modified model generalizes to other samples. Because there were too few boys within each ethnic group to allow testing for measurement invariance of the Dutch MAYSI-2, it is not known whether particular items perform differently across ethnic groups (e.g., something other than the latent trait is involved in the interpretations of the item). It is possible that the model fit indices reported here for the total sample are affected by poor model fit in one of the four ethnic groups. This is an unavoidable caveat of the present study that should be addressed in future research.

When considering several indices together, the internal consistency of the MAYSI-2 scales in the total sample is acceptable to good. However, the reliability indices across the various ethnic groups showed that the Depressed-Anxious and Somatic Complaints (Dutch and Antillean/Surinamese), as well as the Thought Disturbance (Dutch, Antillean/Surinamese, and Mixed) scales were lower than typically acceptable thresholds. Whereas the internal consistency of the MAYSI-2 Though Disturbance is consistently low among most MAYSI-2 studies (Grisso et al. 2012), the reliability indices of MAYSI-2 Depressed-Anxious and Somatic Complaints are most often indicative of acceptable and good internal consistency, respectively, across various ethnic groups (Archer et al. 2004; Grisso et al. 2001). For now, we recommend using these MAYSI-2 scales with caution for clinical decision-making. Future studies are required to test if these scales need revision to increase the reliability of these scales, and whether the need for such a revision may vary across youths from various ethnic origins. Unfortunately, and as was the case for the Dutch MAYSI-2, the internalizing problem scales from other screening tools are also not always as reliable as they ought to be according to commonly used indices (Colins et al. 2013; Vreugdenhil et al. 2006). Therefore, researchers and clinicians may need to accept that brief screens for internalizing problems such as anxiety and depressive feelings may have lower internal consistency, regardless of the instrument being used.

The significant positive correlations between the Dutch MAYSI-2 scales and conceptually similar YSR, SDQ, and DISC-IV-based scales supported the convergent validity of the Dutch MAYSI-2 in the total sample and among Dutch boys. Evidence for divergent validity was less consistent and clear across the scales examined. The well-known comorbidity of mental health problems in detained youths (e.g., Abram et al. 2003) makes it difficult to firmly test the divergent validity of the Dutch MAYSI-2 scales when using YSR and SDQ scales that tap mental health problems that can co-occur (e.g., self-destruction, depressive mood and irritability). Future studies may wish to follow the approach of Archer and colleagues (Archer et al. 2010) and rely on totally different constructs (e.g., medical history of surgery) to test the divergent validity of the Dutch MAYSI-2. Our multivariate regression analyses in part circumvent this comorbidity issue, and provided evidence of divergent validity, as demonstrated by non-significant relations between a Dutch MAYSI-2 scale and conceptually non-parallel YSR or SDQ scales.

Support for the convergent and divergent validity of the Dutch MAYSI-2 scales was less consistent among youths from Moroccan, Antillean/Surinamese and Mixed origins. A possible explanation is that some items in non-conceptually parallel Dutch MAYSI-2 and YSR perform differently across ethnic groups (e.g., different latent meanings), thereby decreasing the likelihood of finding the expected relations. As argued above, testing measurement invariance of the MAYSI-2 is an issue that must be addressed. Despite the YSR and SDQ being used in many countries and cultures, only a few studies have examined measurement invariance, yielding mixed findings across countries (e.g., Lambert et al. 2007) or across ethnic groups within a country (e.g., Richter et al. 2011), and we are aware of no such studies among detained adolescents. Clearly, a lot of work is to be done to ensure several available mental health screening tools can be used and compared across detained youths from various ethnic origins. This to some extent implies that the use of the YSR and SDQ to test the convergent validity of the MAYSI-2 can be considered to be a limitation. Yet, given the lack of other measures that have been shown to be reliable and valid among juvenile justice youths, relying on the YSR and the SDQ is probably the best alternative currently available.

A substantial proportion of detained boys in the U.S. (68 %) and the Netherlands (51 %) score above the caution cut point on one or more MAYSI-2 scales. Conceivably, such elevations suggest the MAYSI-2 to be oversensitive and therefore not clinically useful. However, in the U.S. (Teplin et al. 2002) and the Netherlands (Vreugdenhil et al. 2004), most studies using DSM-based diagnostic tools find that more than 50 % of young people at entry into juvenile detention centers meet criteria for one or more mental disorders. Although the MAYSI-2 is not intended to be diagnostic, the proportion of young people above the cut-offs on MAYSI-2 scales representing mental health symptoms is consistent with the prevalence of psychiatric disorder. If the proportions were lower, one would question the construct validity of the MAYSI-2. Also, referrals for further evaluations can be based on warning cut-offs, which would result in a substantial decrease of identified youths in the present sample. Precisely what criteria should be used to signal further evaluation and what purpose this evaluation has (e.g., avert crisis, comprehensive psychiatric assessment) must be determined by the YDCs’ policy.

### Limitations and Directions for Future Research

The results of this study should be interpreted in the context of several limitations. First, there were too few boys within each ethnic group to test measurement invariance of the Dutch MAYSI-2. Accordingly, differences in mean scores and prevalence of boys at or above MAYSI-2 cut-offs between ethnic group should be interpreted with caution. Nevertheless, in previous studies among detained boys that used the SDQ (Colins et al. 2013) or the YSR (Veen et al. 2010), Moroccan youths had the lowest mean MAYSI-2 scores of the ethnic groups examined. Moroccan youths therefore may systematically report fewer mental health problems than youths from other ethnic origin, regardless of the measure being used. Future studies are warranted to test if this underreport is due to measurement variance issues or whether other factors could explain these cross-ethnic differences.^4^ Notwithstanding this prior recommendation, clinicians likely are much more interested in knowing whether, for example, a Moroccan boy has relatively fewer or greater mental health needs compared with other Moroccan boys, rather than with Dutch of Antillean/Surinamese boys. Consequently, cross-ethnic comparisons may be relatively more relevant for researchers than for clinicians. Second, in the U.S. validation sample, the MAYSI-2 was administered within the first 24 h after admission in 73 % of the cases (Grisso and Barnum 2000). In the current sample, a comparable percentage of youths (i.e., 76 %) filled out the MAYSI-2 within the first 4 days after admission. While the actual detention intake itself may be overwhelming and evoke, for example, anger and depressive feelings, these transient feelings or ‘states’ may decrease over time as a youth adjusts to the situation of being detained. Therefore, the differences in mean scores and percentages of youths at or above MAYSI-2 cut-offs may at least partially be explained by method variance instead of true differences between the U.S. and the present sample. Third, our sample did not include girls. Because the MAYSI-2 in the U.S. has also been validated for use among justice-involved girls (e.g., Grisso and Barnum 2000), future studies are critical to test whether the MAYSI-2 can be used with girls outside the U.S. Finally, in line with many previous papers (e.g., Braam et al. 2010), and because of sample size considerations, Antillean and Surinamese youths were merged together in one group, and Turkish youths, another group of youths who are overrepresented in Dutch YDCs, were included in the Mixed origin group. This approach may have obscured differences regarding the psychometric properties of the MAYSI-2 between these youths and youths from a distinct ethnic origin.

Despite the acknowledge limitations, our study contributes the first empirical investigation of the Dutch MAYSI-2 among a sample of detained adolescents. Our results overall indicate that the translated tool has a similar factor structure as the original measure, good internal consistency across scales, and strong convergent and divergent validity.

## Appendix

**Table 6:** Standardized factor loadings for modified model (44 MAYSI-2 items)

(Scale and item number; Item Description)	Loading
ADU 10. Have you done anything you wish you hadn’t, when you were drunk or high?	0.44
ADU 19. Have your parents or friends thought you drink too much?	0.66
ADU 23. Have you gotten in trouble when you’ve been high or have been drinking?	0.39
ADU 24. If yes, is this fighting?	0.67
ADU 33. Have you used alcohol or drugs to help you feel better?	0.51
ADU 37. Have you been drunk or high at school?	0.72
ADU 40. Have you used alcohol and drugs at the same time?	0.63
ADU 45. Have you been so drunk or high that you couldn’t remember what happened?	0.63
AI 2. Have you lost your temper easily, or had a “short fuse”?	0.45
AI 6. Have you been easily upset?	0.66
AI 7. Have you thought a lot about getting back at someone you have been angry at?	0.88
AI 8. Have you been really jumpy or hyper?	0.55
AI 13. Have you had too many bad moods?	0.73
AI 35. Have you felt angry a lot?	3.97
AI 39. Have you gotten frustrated a lot?	0.52
AI 42. When you have been mad, have you stayed mad for a long time?	0.81
AI 44. Have you hurt or broken something on purpose, just because you were mad?	0.67
DA 3. Have nervous or worried feelings kept you from doing things you want to do?	0.79
DA 14. Have you had nightmares that are bad enough to make you afraid to go to sleep?	0.69
DA 17. Have you felt lonely too much of the time?	0.68
DA 21. Has it seemed like some part of your body always hurts you?	0.56
DA 34. Have you felt that you don’t have fun with your friends anymore?	0.95
DA 35. Have you felt angry a lot?	−3.54
DA 41. Has it been hard for you to feel close to people outside your family?	0.70
DA 47. Have you given up hope for your life?	0.43
DA 51. Have you had a lot of bad thoughts or dreams about a bad or scary event that happened to you?	0.47
SC 27. When you have felt nervous or anxious: have you felt shaky?	0.76
SC 28. When you have felt nervous or anxious: has your heart beat very fast?	0.59
SC 29. When you have felt nervous or anxious: have you felt short of breath?	0.57
SC 30. When you have felt nervous or anxious: have your hands felt clammy?	0.79
SC 31. When you have felt nervous or anxious: has your stomach been upset?	0.61
SC 43. Have you had bad headaches?	0.56
SI 11. Have you wished you were dead?	0.48
SI 16. Have you felt like life was not worth living?	0.67
SI 18. Have you felt like hurting yourself?	0.48
SI 22. Have you felt like killing yourself?	0.72
SI 47. Have you given up hope for your life?	0.18
TD 9. Have you seen things other people say are not really there?	0.59
TD 20. Have you heard voices other people can’t hear?	0.68
TD 25. Have other people been able to control your brain or your thoughts?	0.70
TD 26. Have you had a bad feeling that things don’t seem real, like you’re in a dream?	0.75
TD 32. Have you been able to make other people do things just by thinking about it?	0.66
TE 46. Have people talked about you a lot when you’re not there?	0.48
TE 48. Have you EVER IN YOUR WHOLE LIFE had something very bad or terrifying happen to you?	0.54
TE 49. Have you ever been badly hurt, or been in danger of getting badly hurt or killed?	0.39
TE 51. Have you had a lot of bad thoughts or dreams about a bad or scary event that happened to you?	0.03
TE 52. Have you ever seen someone severely injured or killed (in person – not in movies or on TV)?	0.63

*ADU* = Alcohol/Drug use; *AI* = Angry-Irritable; *DA* = Depressed-Anxious; *SC* = Somatic complaints; *SI* = Suicide ideation; *TE* = Thought disturbance; *TE* = Traumatic experiences

## Footnotes

1 Reliability indices and correlation coefficients that will be presented in the Result section remained substantially similar when youths older than 18 years of age were included (details available upon request from the first author).

2 Specifically, 16 pairs of items were allowed to correlate: items 2 with item 17; item 7 with item 42; item 9 with item 52; item 20 with item 25; item 21 with item 49; item 32 with items 16 and 20; item 40 with item 33; item 41 with items 3, 6, 7, 34, and 47; item 47 with items 3, 34, and 42;

3 Correlations were calculated between all MAYSI-2 scales and the clinical scales of the YSR (*n* = 368), SDQ (*n* = 955) and DISC-IV based Intense Alcohol-Substance Use (*n* = 368).

4 The present study offers little guidance for other explanations, but future research should consider several theoretical possibilities, two of which will be briefly decried here. Arrest patterns may vary across ethnic groups, and detained Moroccans may be more likely to be detained for less serious offenses than Dutch youths (Veen et al. 2010). Therefore, detained Moroccans in the Netherlands may comprise a relatively less seriously disturbed group of youths. Alternatively, detained Moroccans may have had as many mental health needs as detained Dutch youths, but simply were unwilling to report these problems (McCoy 2010), possibly because of concerns about being misunderstood or stigmatized, or due to differences in help seeking behaviour (e.g., seeking other sources of support than mental health professionals) (Bhugra 2004; Lindert et al. 2008)
